# Occurrence of Antimicrobial‐Resistant *Listeria monocytogenes* and *Listeria* spp. Recovered From Cattle Farms and the Factors Associated With Their Distribution in Mpumalanga and North West Provinces in South Africa

**DOI:** 10.1155/vmi/7638995

**Published:** 2026-05-31

**Authors:** K. C. Moabelo, N. C. Mtshali, R. Moerane, N. Gcebe, Y. B. Ngoshe, A. A. Adesiyun

**Affiliations:** ^1^ Department of Production Animal Studies, Faculty of Veterinary Science, University of Pretoria, Private Bag X04 Onderstepoort, Pretoria, 0110, South Africa, up.ac.za; ^2^ Bacteriology Department, Onderstepoort Veterinary Research, Agricultural Research Council, Pretoria, South Africa, arc.agric.za; ^3^ Department of Basic Veterinary Sciences, School of Veterinary Medicine, Faculty of Medical Sciences, University of the West Indies, St. Augustine, Trinidad and Tobago, uwi.edu

**Keywords:** antimicrobial resistance, cattle farms, *Listeria*, mpumalanga, north west, south africa

## Abstract

Two cross‐sectional studies determined the prevalence of antimicrobial resistance (AMR) in *Listeria monocytogenes* and *Listeria* spp. isolated from cattle farms in Mpumalanga and North West provinces in South Africa. A total of 100 isolates comprise 12 and 44 of *L. monocytogenes* and *Listeria* spp., respectively, from Mpumalanga province and 3 and 41 from North West province. The disc diffusion method was used to determine the isolates’ resistance to 16 antimicrobial agents. The studies also investigated the factors (district, farm type, and sample type) associated with AMR. All 100 isolates tested were resistant to at least one antimicrobial agent. Among the isolates of *L. monocytogenes* from Mpumalanga province, the prevalence of resistance ranged from 41.7% (doxycycline) to 100% (cefotaxime, kanamycin, streptomycin, and nalidixic acid), compared to from 33.3% (kanamycin, tetracycline, doxycycline, nalidixic acid, and enrofloxacin) to 100% (streptomycin, nalidixic acid, and ampicillin) in the North West province. Across both provinces, the prevalence of resistance varied significantly for amoxicillin–clavulanate (*p* = 0.0110), kanamycin (*p* = 0.0286), and azithromycin (*p* = 0.0022). Among the *Listeria* spp., resistance ranged from 2.3% (cefotaxime) to 100% (nalidixic acid) in Mpumalanga province, compared to from 14.6% (sulfamethoxazole–trimethoprim, SXT) to 100% (nalidixic acid) in North West. For *L. monocytogenes* and *Listeria* spp. from both provinces, resistance to antimicrobial class was low for SXT (6.0%) and β‐lactams (9.7%) but comparatively high for lincosamides (50%) and fluoroquinolone (62.3%) (*p* < 0.00001). Of the three factors (district, farm type, and sample type) investigated for their association with the prevalence of resistance in *L. monocytogenes* and *Listeria* spp. in both provinces, the district location of the farm was the only factor that statistically significantly (*p* = 0.0455) affected the prevalence of resistance in *L. monocytogenes* to ciprofloxacin in only Mpumalanga province. Notably, on a larger geographic scale, Mpumalanga and North West provinces showed significant (*p* < 0.05) differences in the prevalence of ampicillin resistance. The high prevalence of resistance to multiple antimicrobial agents across sources and the two provinces may pose therapeutic challenges for treating listeriosis on cattle farms.

## 1. Introduction

South Africa experienced the largest outbreak of human listeriosis in 2017–2018, in which 216 deaths and more than 1060 laboratory‐confirmed cases were documented across the country’s nine provinces [1, 2]. The outbreak, which lasted for approximately 18–19 months, was managed with antimicrobial agents to reduce the risk of additional mortality and morbidity. According to NICD [3], the clinical isolates were highly susceptible to ampicillin, which remained the recommended first‐line treatment, often in combination with gentamicin. The importance of having effective treatments available for human and animal listeriosis cannot, therefore, be ignored.

Listeriosis in humans, primarily caused by *L. monocytogenes*, is associated with severe clinical manifestations such as abortion, preterm birth, or stillbirth in pregnant women [4, 5], meningitis or encephalitis, and death [4, 6, 7]. In animals, listeriosis has been reported to cause encephalitis, abortion, mastitis, repeat breeding, and endometriosis, causing significant economic production losses [8, 9].

The use of antibiotics to control clinical listeriosis has been documented to contribute to the selection of resistant strains, particularly for antibiotics commonly used to treat listeriosis. β‐Lactams (penicillin and ampicillin), with or without gentamicin, are considered the main antibiotics for the treatment of listeriosis [4, 10–12]. It has also been recommended that vancomycin and trimethoprim–sulfamethoxazole can be used as alternative therapy for penicillin‐allergic patients [13, 14]. Veterinary use of antimicrobial agents in food‐producing animals, such as cattle, which are commonly administered for disease therapy, prophylaxis, and growth promotion, has been reported to contribute to the emergence of resistant strains [15, 16], with production and economic implications. The practice of using antimicrobial agents in livestock feeds as growth promoters has reduced their efficacy, thereby diminishing their impact on infectious diseases [17]. Furthermore, reduced antimicrobial effectiveness has led to the spread of antimicrobial‐resistant *L. monocytogenes*, which is associated with the presence of a plasmid or with genes transferred by several mechanisms, including conjugation, transposition, nonantibiotic stressors, and biofilms [18, 19]. It has therefore been recognized that antimicrobial resistance (AMR) poses a public health concern, as it not only limits treatment options but also causes economic problems globally [20]. The complications associated with resistant *L. monocytogenes* and other pathogens in humans and animals cannot be overemphasized.

High prevalence of AMR, including *L. monocytogenes*, has been reported to be over 90% resistance to ampicillin and erythromycin on cattle farms in Jordan [21] and resistance to penicillin (100%) and amoxicillin (100%) in *Listeria* spp. isolated from cattle feces in Dhaka [22], while in the United States, 86%, 94.8%, and 89.6% of isolates of *L. monocytogenes* recovered from dairy cattle manure amended farms were resistant to kanamycin, nalidixic acid, and levofloxacin, respectively [23].

In the beef production chain, antimicrobial agents may vary in type and frequency of use, potentially reducing their effectiveness. The problem is compounded in developing countries, where documented reports of uncontrolled antimicrobial use have led to a high prevalence of AMR [24, 25]. Furthermore, some variables or factors, such as the geographical location of cattle farms, the type of farms and practices, and the types of samples, have been documented to affect the prevalence of AMR [26–29].

Globally, the trend is to implement the One Health approach to control AMR in *Listeria*, which involves coordinating surveillance, hygiene, and antibiotic stewardship across human, animal, and environmental sectors [30–33]. This approach focuses on reducing agricultural antibiotic use, improving food safety protocols to prevent environmental contamination, and monitoring transmission pathways to manage risks [34, 35]. It cannot be overemphasized that the One Health approach must be implemented holistically to be successful.

Therefore, in South Africa, in response to the increased threat of AMR, the government developed a five‐strategic framework for national plans, which includes early detection and the optimization of AMR surveillance to report national, regional, and local resistance patterns for targeted antimicrobials [36, 37]. It is pertinent to note that, although the framework’s focus was on the One Health approach, implementation encountered several challenges. This is because the country, as in most developing countries, has policies on antimicrobial types and use; however, enforcement and control of their use remain major hindrances, particularly due to inadequate personnel [38]. Additionally, the government of South Africa introduced the Fertilizers, Farm Feeds, Agricultural Remedies, and Stock Remedies Act 36 of 1947, which legalized the over‐the‐counter (OTC) availability of specific antimicrobial agents to facilitate the timely treatment of readily identifiable endemic diseases. This initiative was in response to the challenges faced by communal livestock farmers, including high disease incidence compounded by poor veterinary extension services [39–41]. The outcome of this policy is that the least expensive OTC antimicrobial agents are widely used without veterinary oversight [42, 43]. Across the country, phenotypic methods have reported varying frequencies of AMR *L. monocytogenes* and *Listeria* spp. isolated from meat and meat products [44], ready‐to‐eat foods [45, 46], clinical cases, and the environment in the Western Cape [47], fruits and vegetables in the Eastern Cape [48], beef and beef products from retail outlets in Gauteng province [49] and in North West province [50], and fresh produce in KwaZulu‐Natal Province [51]. To date, no information is available on the antibiograms of *L. monocytogenes* and *Listeria* spp. recovered from cattle farms in Mpumalanga and North West provinces in South Africa, nor are there data available on the factors that may affect their distribution. Furthermore, to obtain an updated database in the country, it was imperative to investigate the AMR status in *L. monocytogenes* in Mpumalanga and North West provinces, based on the documentation of cases and deaths in the world’s largest human listeriosis outbreak in the country [1, 2] and the comparatively high cattle population in the country [52]. The need to implement a holistic approach to address the widespread high prevalence of AMR in *L. monocytogenes* and possibly other bacteria in cattle and other livestock, as the resistant strains have the potential to enter the human food chain.

Therefore, the current study investigated and compared the prevalence of resistance to antimicrobial agents, including those used in cattle and humans, in *L. monocytogenes* and *Listeria* spp. isolated from cattle farms in Mpumalanga and North West provinces in South Africa. The study also examined the potential association of three factors (district, farm type, and sample type) with the occurrence of AMR to 16 antimicrobial agents across eight classes in *L. monocytogenes* and *Listeria* spp.

## 2. Materials and Methods

### 2.1. Study Design

The study was conducted to determine the prevalence of AMR among *L. monocytogenes* and other *Listeria* spp. recovered from many sources on cattle farms and to assess the factors associated with their distribution in Mpumalanga and North West provinces in South Africa.

To conduct these cross‐sectional studies, the required sample size was estimated using the formula recommended by Thrusfield [53], with a prevalence of *L*. *monocytogenes* and *Listeria* spp. of *P*_exp_ value of 50% and a *d*‐value of 4.5%, resulting in an estimated sample size of 480; however, in the Mpumalanga province study, a total of 475 were obtained, while in the North West province study, 204 were obtained.

In South Africa, cattle farms play a vital role in the beef production chain. There are three categories of cattle farming: communal farms, cow–calf operations, and feedlots. The operations of these categories of cattle farms in the country have been described earlier [54].

### 2.2. Selection of Cattle Farms, Sources, Types, and Collection of Samples, and Transportation to the Laboratory for Processing

Information on the types and locations of cattle farms in both provinces was obtained from the Department of Agriculture [37]. The study design was to randomly select farms from farmers who agreed to participate. The design was to collect samples from each of the two provinces, namely, feedlots (*n* = 5), cow–calf operations (*n* = 10), and communal farms (*n* = 10). These three categories of farms were available for sampling in Mpumalanga province. However, in the North West province, none of the feedlot operations approached agreed to participate in the study, citing the risk of their workers being exposed to COVID‐19.

### 2.3. Locations of Cattle Farms in Mpumalanga and North West Provinces According to the Districts

**FIGURE 1:**
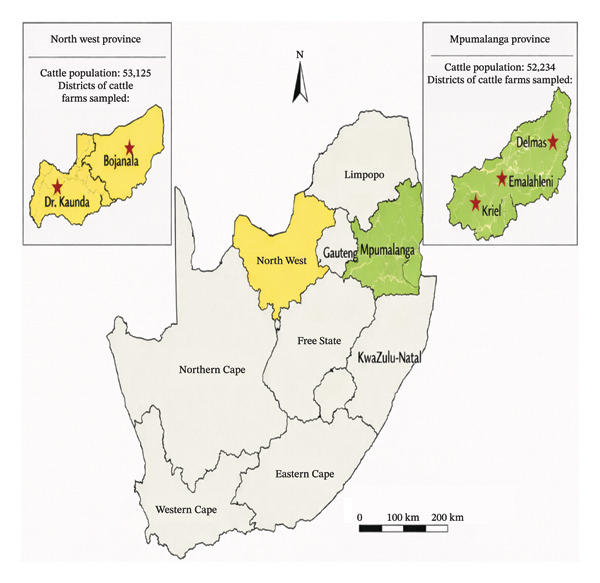
Map of Mpumalanga and North West provinces.

Figure 1 is a map of South Africa, including Mpumalanga and North West provinces, and the district locations of the cattle farms sampled for the study. The cattle population of 52,234 and 53,125 in Mpumalanga and North West provinces, respectively, was also displayed. The total cattle population across the nine provinces of South Africa in the 2022 animal census was 565,444 [52].

### 2.4. Questionnaire Administration

Questionnaires were administered to farm owners or managers at cattle farms where the study was conducted to collect demographic data and identify risk factors for *L. monocytogenes* infection. The questionnaire was administered to capture demographic data, such as the district, farm name, global positioning system (GPS) coordinates, type of farm (feedlots, cow–calf operation, and communal), farm animal population, animal information (age, sex, breed) and history of animals sampled (experience of listeriosis, farm bred/purchased), and management practices.

### 2.5. Sample Collection From Cattle Farms in Mpumalanga and North West Provinces

The details of how the samples (feces from individual cattle and freshly voided pooled feces, drinking water, effluent, feeds [grass, grain], and silage) were collected were provided in our earlier study [55].

Specifically for cattle‐related feces (rectal feces grabs or freshly voided feces from individual cattle), a long‐arm glove was used to collect samples from the rectum of individually selected cattle. Pooled fecal samples are freshly voided feces collected from areas where cattle frequently congregate, such as feeding or resting areas. Samples were collected using hand gloves or wooden spatulas from portions of the freshly voided feces that had not made direct contact with the ground or floor.

### 2.6. Demographic Data Analysis of Samples Collected From Cattle Farms in Mpumalanga and North West Provinces

A total of 475 samples were collected at 25 farms across three districts in Mpumalanga province, South Africa. Of the 475 samples collected, the distribution within the levels of the three variables (district, type of farm, and type of samples) studied was statistically significantly different (*p* < 0.05) (Table 1). The highest number of samples collected in each category was 46.9% (223/475, 95%CI: 42.5–51.5), 40.0% (190/475, 95%CI: 35.9%–44.7%), and 48.4% (230/475, 95%CI: 43.9–52.9) for district (Emalahleni), farm type (feedlot operations), and sample type (individual cattle feces), respectively. The sources of the 12 and 44 isolates of *L. monocytogenes* and *Listeria* spp., respectively, were previously documented [55]. However, their antibiograms were determined in the current study.

**TABLE 1:** Distribution of samples collected from Mpumalanga and North West provinces according to the districts, the type of farm, and the type of sample.

Province	Variable	Level	No. of samples	Percentage		*p* value
				Rate (%)	95%CI	
Mpumalanga	District	Delmas	199	41.9	37.5–46.4	0.00001
		Emalahleni	223	46.9	42.5–51.5	
		Kriel	53	11.2	8.6–14.3	
	Type of farm	Communal	120	25.3	21.5–29.4	0.0254
		Cow–calf operation	165	34.7	30.4–38.9	
		Feedlot	190	40	35.9–44.7	
	Type of sample	Effluent	30	6.3	4.4–8.9	< 0.0001
		Individual feces^a^	230	48.4	43.9–52.9	
		Pooled feces^b^	55	11.6	8.9–14.8	
		Feeds	85	17.9	14.7–21.6	
		Silage	30	6.3	4.4–8.9	
		Water	45	9.5	7.1–12.5	

North West	District	Bojanala Platinum	94	46.1	39.4–52.9	0.2611
		Dr. Kenneth Kauda	110	53.9	47.1–60.6	
	Type of farm^c^	Cow–calf	124	60.8	54.1–67.5	0.0024
		Communal	80	39.2	32.5–45.9	
	Type of sample^d^	Feces	176	86.3	81.5–91.0	< 0.0001
		Feed	10	4.9	1.9–7.9	
		Water	18	8.8	4.9–12.7	

^a^Feces freshly collected from the individual cattle’s rectum or from freshly voided feces.

^b^Feces pooled from areas where cattle congregate to eat feeds and drink water in the troughs eat feeds and drink water in troughs.

^c^All feedlot operations in the province declined participation in the study because of the prevailing national health emergency at the time of the study. Therefore, no feedlot operation was available for sampling for the study.

^d^None of the cattle farms had effluents during the visits to collect samples.

A total of 204 samples were collected in the North West province from the randomly selected cattle farms, comprising 10 each of communal and cow–calf operations. In the study, samples were collected from two districts: Bojanala Platinum (94; 46.1%) and Dr Kenneth Kaunda (110; 53.9%). The highest frequency of samples collected in each category was 53.9% (95% CI: 47.1%–60.6%), 60.8% (95% CI: 54.1%–67.5%) and 86.3% (95% CI: 81.5%–91.0%) for the district (Dr. Kenneth Kauda), farm type (cow–calf), and sample type (feces), respectively.

The challenges posed by farmers’ reluctance to participate in the studies were attributable to the COVID‐19 pandemic in the country during the study period, which accounted for the significant differences in the number of samples collected across variables and provinces. The details of the type of samples (pooled feces, drinking water, feeds, silage, effluents, and wastewater) and collection methods were earlier described [49, 55, 56].

All samples from the farms were transported to the ARC‐Onderstepoort Veterinary Institution Feed and Food Laboratory within 12 h of collection and processed within 48 h.

### 2.7. Enrichment, Isolation, and Identification of *L. monocytogenes* and *Listeria* spp.

The methodology used to enrich, isolate, and confirm *L. monocytogenes* and *Listeria* spp. was reported in our earlier study [55].

### 2.8. Enrichment of Samples Collected

To isolate and identify *L. monocytogenes* and *Listeria* spp., standard bacteriological methods and polymerase chain reaction (PCR) were used to analyze the samples, as previously described [57, 58].

All samples collected from cattle and farm environments were subjected to an enrichment process. For feces, feeds, and silage samples, they were aseptically removed from their cups, weighed using a weighing balance, and 10 g was transferred aseptically into a stomacher bag containing 90 mL of ONE Broth‐*Listeria* (Thermo Fisher Scientific, South Africa). This was followed by homogenization and aerobic incubation at 35°C for 48 h. For effluent and drinking water samples, the water centrifugation method was used. For each sample, 100 mL was aliquoted into four 25 ‐mL portions in centrifuge tubes and then spun down at 13,000  ×  g for 5 min. The pellets from the four bottles were pooled and inoculated into 9 mL of ONE Broth*‐Listeria* (Thermo Fisher Scientific, South Africa) for enrichment, followed by aerobic incubation at 35°C for 48 h. The enriched sample broth was used to inoculate Brilliance *Listeria* agar (BLA) (Thermo Fisher Scientific, South Africa) plates to isolate *Listeria* spp.

### 2.9. Isolation of *L. monocytogenes* and *Listeria* spp. From Enriched Samples in Mpumalanga and North West Provinces

A loopful of enriched broth culture was inoculated onto ONE Broth‐*Listeria* (Thermo Fisher Scientific, South Africa) and streaked for isolation on BLA plates, followed by 48‐h incubation at 35°C. *Listeria* spp. and *L. monocytogenes* were phenotypically confirmed based on the characteristic colony morphology on BLA. *Listeria* spp. appeared as blue colonies without a halo, while *L. monocytogenes* appeared as blue colonies with a white/cream halo [55, 57, 59]. Single colonies of suspected *Listeria* spp. and *L. monocytogenes* were subcultured on BLA for further purification.

### 2.10. Molecular Identification and Characterization of *Listeria* spp. and *L*. *monocytogenes*

#### 2.10.1. Screening of Suspect Isolates of Listeria spp. by Conventional PCR

All enriched broth samples were screened by conventional PCR for *Listeria* spp., i.e., the *Listeria* genus.

DNA was extracted using the boiling–centrifugation method described by Soumet et al. [60]. Briefly, aliquots (2 mL) of enrichment broth were spun at 13,000  ×  g for 5 min in a centrifuge (Eppendorf, South Africa). The pellets were suspended in 200 μL of sterile bidistilled water, heated to 95°C in a dry block for 10 min, cooled at room temperature for 5 min, and centrifuged at 13,000  ×  g for 5 min. The supernatant was pipetted into sterile tubes, and the pellet was discarded. The DNA in the supernatant was then used for further characterization using PCR.

Screening by PCR was performed using a multiple PCR (mPCR) assay targeting the *prs* gene (a *Listeria* genus–specific gene), as previously described by Doumith et al. [61]. The following primers were used: *ORF2110, ORF2819, Imo*1118, *Imo0737*, and *Prs.* The PCR products were subjected to electrophoresis on a 3% agarose gel for 3 h at 120 V. *L. monocytogenes* ATCC 19111 was used as a positive control, and water was used as a negative control.

Overall, in Mpumalanga province, a total of 56 isolates, comprising 12 and 44 of *L. monocytogenes* and *Listeria* spp., respectively, were confirmed and tested for AMR. In the North West province, a total of 44 isolates, comprising 3 and 41 isolates of *L. monocytogenes* and *Listeria* spp., respectively, were confirmed and tested for AMR. In both provinces, 100 *Listeria* isolates were confirmed, comprising 15 *L. monocytogenes* and 85 *Listeria* spp.

### 2.11. Determination of the Susceptibility of *L. monocytogenes* and Other *Listeria* spp. to Antimicrobial Agents

The 16 antimicrobial agents selected for the study were chosen based on their availability to livestock farmers in South Africa and their use by veterinarians and human medical practitioners to treat infections caused by *L. monocytogenes* and other pathogenic bacteria in the country. The classes and concentrations of the 16 antimicrobial agents are shown in Table 2. The susceptibility of the isolates of *L. monocytogenes* and *Listeria* spp. recovered from cattle farms in Mpumalanga and North West provinces was phenotypically tested against 16 antimicrobial agents. The Kirby–Bauer disc diffusion method was used in accordance with the Clinical and Laboratory Standards Institute (CLSI) guidelines and interpretations [62]. For the study, we used *L. monocytogenes* ATCC 18111, *Listeria innocua* ATCC 33090, and *Campylobacter jejuni* ATCC 273373 as controls. The inhibition zones were classified as susceptible (S) or resistant (R) to the tested antimicrobial agents. However, for antimicrobial agents for which cutoff values for *Listeria* susceptibility were not provided, staphylococcal cutoff values were used, as recommended by Conter et al. [63].

**TABLE 2:** List of 16 antimicrobial agents in eight classes used for the isolates of *Listeria monocytogenes and Listeria* spp.

Antimicrobial class	Antimicrobial agent	Concentration (*μ*g)
Beta‐lactam	Penicillin	10
	Amoxicillin–clavulanic acid	30
	Ampicillin	10

Cephalosporins	Cephalothin	30
	Cefotaxime	30

Aminoglycosides	Streptomycin	25
	Gentamicin	10
	Kanamycin	30

Tetracyclines	Tetracycline	30
	Doxycycline	30

Fluoroquinolones	Nalidixic acid	30
	Ciprofloxacin	5
	Enrofloxacin	5

Macrolides	Azithromycin	15

Lincosamides	Clindamycin	10

Sulfonamides	Sulfamethoxazole–trimethoprim (SXT)	23.75/1.25

### 2.12. Data Analysis

The Statistical Package for Social Sciences (SPSS) and Epi Info were used to generate prevalence, frequency, and percentage data on AMR in *L. monocytogenes* and *Listeria* spp. according to the variables investigated: province (Mpumalanga and North West), among multiple districts (Delmas, Emalahleni, and Kriel; Bojanala Platinum and Dr. Kaunda), types of farms (communal, cow‐calf, feedlot), and types of sample (effluent, feces, feed, silage, and drinking water). For each of the 16 antimicrobial agents, a 2 × 2, (3 × 2), (4 × 2), or (5 × 2) contingency table was constructed for comparing resistant versus nonresistant isolates between the two groups. The data were entered into Microsoft Excel 2016 and analyzed. The data were analyzed using R and Stata 15, and the association between the variables and the occurrence of AMR in the isolates was assessed using Fisher’s exact and chi‐square tests. Fisher’s exact test (two‐tailed) was used where at least one expected cell count was < 5 in several comparisons, and the chi‐square test was used where all expected counts were ≥ 5. The significance level was set at 0.05.

## 3. Results

### 3.1. AMR of *L. monocytogenes* and *Listeria* spp. Isolated From Farms in Mpumalanga Province

**FIGURE 2:**
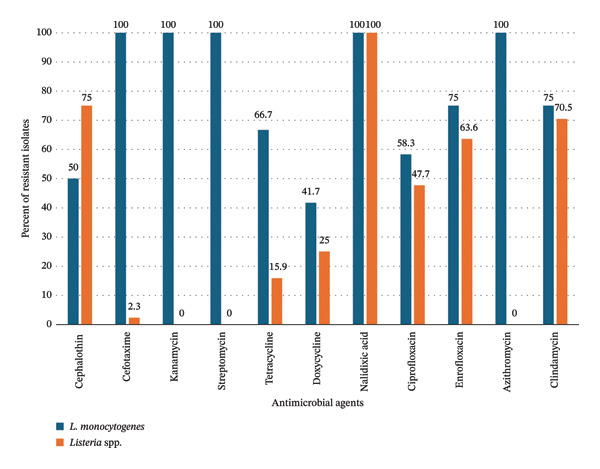
Prevalence of AMR in Mpumalanga province.

The prevalence of resistance to antimicrobial agents in *L. monocytogenes* isolates recovered from cattle farms in Mpumalanga province is shown in Figure 2. Of the 16 antimicrobial agents, the 12 isolates of *L. monocytogenes* were resistant to 11 (68.8%) but susceptible to penicillin, ampicillin, amoxicillin–clavulanic acid, sulfamethoxazole–trimethoprim, and gentamycin. The prevalence of resistance ranged from 41.7% (5/12, 95% CI: 15.2–72.3) for doxycycline to 100% (12/12, 95% CI: 73.5–100.0) for cefotaxime, kanamycin, streptomycin, nalidixic acid, and azithromycin. The difference was statistically significant (*p* < 0.05).

Among isolates of other *Listeria* spp., resistance was observed to 8 (50.0%) of the 16 antimicrobial agents tested. For the eight antimicrobial agents to which resistance was exhibited, all (100.0%) of the 44 isolates were resistant to nalidixic acid (Figure 2). The prevalence ranged from 2.3% (1/44, 95% CI: 0.1–12.0) for cefotaxime to 100% (44/44, 95% CI: 91.7–100.0) for nalidixic acid. The difference was statistically significant (*p* < 0.05). The eight antimicrobial agents to which all the *Listeria* spp. isolates were susceptible to penicillin, ampicillin, amoxicillin–clavulanic acid, sulfamethoxazole–trimethoprim, gentamycin, kanamycin, streptomycin, and azithromycin.

For the eight antimicrobial agents to which *L. monocytogenes* and *Listeria* spp. exhibited resistance, statistically significant differences were observed with cefotaxime (*L. monocytogene*s: 100.0%, *Listeria* spp.: 2.3%, *p* < 0.0001) and tetracycline (*L. monocytogenes*: 66.7%, *Listeria* spp.: 15.9%, *p* = 0.0004). Furthermore, the prevalence of resistance to antimicrobial agents was higher in *L. monocytogenes* than in *Listeria* spp. for cephalothin, doxycycline, ciprofloxacin, enrofloxacin, and clindamycin. However, the differences were not statistically significant (*p* > 0.05).

A comparison of the frequency of resistance to the 16 agents tested in *L. monocytogenes* (*n* = 12) was 68.8% (11/16) compared with the 50% (8/16) found in *Listeria* spp. (*n* = 44). The difference was not statistically significant (*p* = 0.2802). Susceptibility to five antimicrobial agents (penicillin, ampicillin, amoxicillin–clavulanic acid, gentamycin, and sulfamethoxazole–trimethoprim) was common to both *L. monocytogenes* and *Listeria* spp. in Mpumalanga province.

### 3.2. AMR of *L. monocytogenes* and *Listeria* spp. Isolated From Farms in North West Province

**FIGURE 3:**
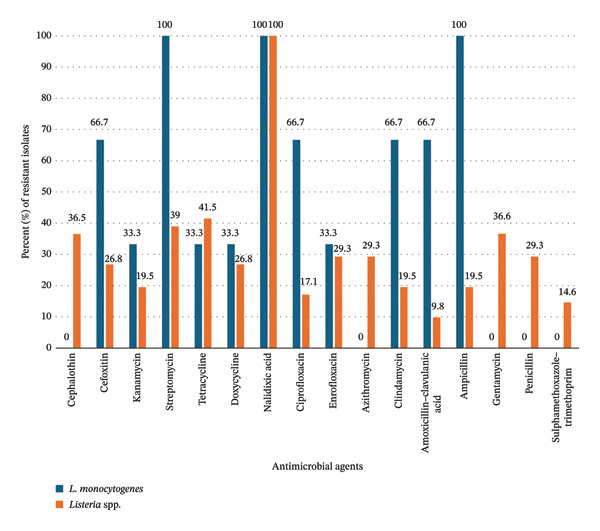
Prevalence of AMR in L. monocytogenes and Listeria spp. from North West province.

The 3 *L. monocytogenes* isolates and 41 other *Listeria* spp. (other than *L. monocytogenes*) recovered from cattle farms in the North West province, all exhibited resistance to more than one of the 16 antimicrobial agents assessed in the study (Figure 3). The differences in resistance prevalence to the 16 antimicrobial agents were significant (*p* < 0.05).

For the antimicrobial agents to which resistance was exhibited by *L. monocytogenes*, the prevalence of resistance ranged from 33.3% (kanamycin, tetracycline, doxycycline, and enrofloxacin) to 100% (streptomycin and nalidixic acid). The differences were statistically significant (*p* < 0.05). All 3 *L. monocytogenes* isolates were susceptible to five antimicrobial agents (cephalothin, azithromycin, gentamycin, penicillin, and sulfamethoxazole–trimethoprim).

Among the 41 isolates of *Listeria* spp., resistance was shown to 16 (100%) antimicrobial agents (100%) at a range from 9.8% (amoxicillin–clavulanic acid) to 100% (nalidixic acid (Figure 3)).

A comparison of the prevalence of resistance exhibited by *L. monocytogenes* (*n* = 3) and *Listeria* spp. (*n* = 41) in North West province revealed a statistically significantly higher prevalence in *L. monocytogenes* compared to *Listeria* spp., to three antimicrobial agents as follows: ciprofloxacin (*L. monocytogenes*: 66.7% versus *Listeria* spp.: 17.1%, *p* = 0.0398), amoxicillin–clavulanic acid (*L. monocytogenes*: 66.7% versus *Listeria* spp.: 9.8%, *p* = 0.0056), and ampicillin (*L. monocytogenes*: 100% versus *Listeria* spp.: 19.5%, *p* = 0.0212).

### 3.3. Investigation of the Effect of Geographic Locations (Mpumalanga and North West Provinces) on the Prevalence of Resistance to Antimicrobial Agents in *L. monocytogenes* and *Listeria* spp.

A comparison of the prevalence of resistance to antimicrobial agents in *L. monocytogenes* recovered from cattle farms in two geographically different provinces, Mpumalanga (*n* = 12) and North West (*n* = 3), revealed that the resistance prevalence was the same in both provinces: 0.0% for three antimicrobial agents (penicillin, gentamycin, and sulfamethoxazole–trimethoprim). However, a statistically significantly higher prevalence of resistance to two therapeutically important agents for treating listeriosis was detected in the North West province compared with Mpumalanga province to Amoxicillin‐clavulanic acid (Mpumalanga: 0.0% versus North West: 66.7%; *p* = 0.011) and to Ampicillin (Mpumalanga: 0.0% versus North West: 100%; *p* = 0.0022). The reverse was the case with two antimicrobial agents, where statistically higher prevalence of resistance was found in isolates of *L. monocytogenes* from Mpumalanga province than found in North West isolates: kanamycin (Mpumalanga province: 100% versus North West province: 33.3%, *p* = 0.0286), and azithromycin (Mpumalanga province: 100% versus North West province: 0.0%, *p* = 0.0022).

### 3.4. Prevalence of Resistance to Antimicrobial Agents by *L. monocytogenes* and *Listeria* spp. From Mpumalanga and North West Provinces by the Class of Antimicrobial Agents

Table 3 presents the prevalence of resistance to 16 antimicrobial agents across the eight classes in *L. monocytogenes* and *Listeria* spp. pooled from Mpumalanga and North West provinces. Among *L. monocytogenes* isolates (*n* = 15), resistance was detected in seven (87.5%) of eight antimicrobial classes; none were resistant to the sulfamethoxazole–trimethoprim class. The prevalence of resistance ranged from 33.3% (beta‐lactams) to 100% (aminoglycosides, tetracyclines, and fluoroquinolones), and the difference was statistically significant (*p* = 0.0025).

**TABLE 3:** Prevalence of resistance to antimicrobial agents by *L. monocytogenes* and *Listeria* spp. from Mpumalanga and North West provinces by class of antimicrobial agents.

Antimicrobial class (8)	Type of antimicrobial agent	No. (%) of isolates that exhibited resistance^a^ to antimicrobial agents in class:			
		*L. monocytogenes* (*n* = 15)^b^	*Listeria* spp. (*n* = 85)^c^	*p* value	Total (*n* = 100)
β‐Lactams (3)	Penicillin, amoxicillin, clavulanic acid, ampicillin	5 (33.3)	12 (14.1)	0.0776	17 (17.0)
Cephalosporins (2)	Cephalothin, cefotaxime	14 (93.3)	60 (70.6)	0.0641	74 (74.0)
Aminoglycosides (3)	Streptomycin, gentamycin, kanamycin	15 (100.0)	39 (45.9)	0.0001	54 (54.0)
Tetracyclines (2)	Tetracycline, doxycycline	15 (100.0)	46 (54.1)	0.0003	61 (61.0)
Fluoroquinolones (3)	Nalidixic acid, ciprofloxacin, enrofloxacin	15 (100.0)	85 (100.0)	NA	100 (100.0)
Macrolides (1)	Azithromycin	12 (80.0)	12 (14.1)	< 00001	24 (24.0)
Lincosamides (1)	Clindamycin	11 (73.3)	39 (45.9)	0.050	52 (52.0)
Sulfonamides (1)	Sulfamethoxazole–trimethoprim (SXT)	0 (0.0)	6 (7.1)	0.5872	6 (6.0)
*p* value		0.0025	< 0.00001		< 0.00001

^a^Resistant to any of the antimicrobial agents in the class.

^b^Consisting of 12 and 3 *L. monocytogenes* from Mpumalanga and North West provinces, respectively.

^c^Comprising 44 and 41 *L. monocytogenes* from Mpumalanga and North West provinces, respectively.

Among the 85 isolates of Listeria spp. resistant to antimicrobial agents in the eight antimicrobial classes, resistance ranged from 7.1% (sulfamethoxazole–trimethoprim) to 100% (nalidixic acid). The differences were statistically significant (*p* <0.0001)

For a comparison of resistance between *L. monocytogenes* and *Listeria* spp. according to the antimicrobial classes, there was a statistically significant difference in four antimicrobial classes as follows: aminoglycosides (*L. monocytogenes*, 100% versus *Listeria* spp., 45.t9%; *p* = 0.0001), tetracyclines (*L. monocytogenes*, 100% versus *Listeria* spp., 54.1%; *p* = 0.0003), macrolides (*L. monocytogenes,* 80% versus *Listeria* spp., 14.1%; *p* < 0.0001), and lincosamides (*L. monocytogenes*, 73.3% versus *Listeria* spp., 45.9%; *p* < 0.050).

According to the total number of isolates of both *L. monocytogenes* and *Listeria* spp., the prevalence of resistance by the antimicrobial class was comparatively low for sulfonamides (6%), beta‐lactam (17%), and macrolides (24%) but high for tetracyclines (61%), cephalosporins (74%), and fluoroquinolones (100%). The differences were statistically significantly different (*p* < 0.00001).

### 3.5. Prevalence of AMR *L. monocytogenes* and *Listeria* spp. Isolates From Mpumalanga Province According to the District

The prevalence of resistance to antimicrobial agents by *L. monocytogenes* and *Listeria* spp. isolates by district in Mpumalanga province is shown in Table 4. *L. monocytogenes* was isolated from only two districts (Delmas and Emalahleni) to be tested for resistance to antimicrobial agents in the current study. AMR was high (50%–100%) for three antimicrobial agents (CEF, NA, and CLIN) constituting 37.5% of eight agents to which resistance was found in Delmas province, while in Emalahleni, resistance ranging from of 50%–100% was detected in six agents (TE, DOX, NA, CIP, ENR, and CLIN), representing 75% of the eight agents for which resistance was exhibited. An analysis of the prevalence of resistance to eight antimicrobial agents across the two districts revealed a statistically significant difference for ciprofloxacin only (Delmas: 0.0% versus Emalahleni: 77.8%; *p* = 0.0455).

**TABLE 4:** Antimicrobial resistance of isolates of *L. monocytogenes* and *Listeria* spp. recovered from farms in Mpumalanga province to 16 antimicrobial agents, according to the district.

District^a^	No. of isolates of *L. monocytogenes* tested^b^	No. (%) of isolates resistant to:							
		CEF	CEP	TE	DOX	NA	CIP	ENR	CLIN
Delmas	3	2 (66.7)	0 (0.0)	1 (33.3)	0 (0.0)	3 (100.)	0 (0.0)	1 (33.3)	3 (100)
Emalahleni	9	4 (44.4)	0 (0.0)	7 (77.8)	5 (55.6)	9 (100.0)	7 (77.8)	8 (88.9)	6 (66.7)
*P* value		0.5050	NA	0.1573	0.2045	NA	0.0455	0.0543	0.5091
Subtotal	12	6 (50.0)	0 (0.0)	8 (66.7)	5 (41.7)	12 (100.0)	7 (58.3)	9 (75.0)	9 (75.0)

**District**	**No. of isolates of L*isteria* spp. tested**	**CEF**	**CEP**	**TE**	**DOX**	**NA**	**CIP**	**ENR**	**CLIN**

Delmas	14	0 (0.0)	11 (78.6)	4 (28.6)	5 (35.7)	14 (100.0)	6 (42.9)	11 (78.6)	13 (92.9)
Emalahleni	26	1 (3.9)	18 (69.2)	2 (7.7)	6 (23.1)	27 (100.0)	12 (46.1)	15 (57.7)	15 (57.7)
Kriel	4	0 (0.0)	4 (100.0)	1 (25.0)	0 (0.0)	4 (100.0)	3 (75.0)	2 (50.0)	3 (75.0)
*p* value		1.0	0.5504	0.1982	0.2778	1.0	0.5088	0.3556	0.0656
Subtotal	44	1 (2.3)	33 (75.00)	7 (15.9)	11 (25.0)	44 (100.0)	21 (47.7)	28 (63.6)	31 (70.5)
Total	56	7 (12.5)	33 (58.9)	15 (26.8)	16 (28.6)	56 (100.0)	28 (50.0)	37 (66.1)	40 (71.4)

^a^All the isolates of *L. monocytogenes* and *Listeria* spp. were susceptible to penicillin, amoxicillin–clavulanic acid, ampicillin, sulfamethoxazole–trimethoprim, kanamycin, gentamycin, streptomycin, and AZI: azithromycin.

^b^No *L. monocytogenes* isolates [55] were available for AMR testing.

Among *Listeria* spp., the prevalence of resistance ranged from 50% to 100% in four agents (CEF, NA, ENR, and CLIN), in four agents (CEP, NA, ENR, and CLIN), and in five agents (CEP, NA, CIP, ENR, and CLIN) in Delmas, Emalahleni, and Kriel districts, respectively. Similar patterns of high resistance were observed in both Delmas and Emalahleni. Overall, the differences in the prevalence of resistance among *Listeria* spp. to the eight antimicrobial agents across the three districts were not statistically significant (*p* > 0.05).

### 3.6. Prevalence of AMR *L. monocytogenes* and *Listeria* spp. Isolates From the North West Province According to the District

The 3 *L. monocytogenes* isolates originated from a single district (Dr. Kaunda); therefore, no comparisons by district were possible (Table 5). Overall, across the 16 antimicrobial agents tested, the prevalence of resistance in the range of 20%–40% is low, with the detection in five antimicrobial agents (AMP, K, TE, DOX, and ENR), representing 31.2% of the 16 agents. For the 41 *Listeria* spp. recovered from the two districts, although resistance was detected across the 16 antimicrobial agents, the differences were not statistically significant (*p* > 0.05).

**TABLE 5:** Prevalence of antimicrobial resistance of *L. monocytogenes and Listeria* spp. was recovered from cattle farms according to the districts of North West province.

District	No. of *L. monocytogenes* isolates tested^a^	No. (%) of isolates resistant to:															
		P	AMC	AMP	CEP	CEF	S	CN	K	TE	DOX	NA	CIP	ENR	CLIN	SXT	AZM
Dr. Kaunda	3	0 (0.0)	2 (66.7)	1 (33.3)	0 (0.0)	2 (66.7)	3 (100.0)	0 (0.0)	1 (33.3)	1 (33.3)	1 (33.3)	3 (100.0)	2 (66.7)	1 (33.3)	2 (66.7)	0 (0.0)	0 (0.0)

**District**	**No. of *Listeria* spp. isolates tested^b^**	**P**	**AMC**	**AMP**	**KF**	**CTX**	**S**	**CN**	**K**	**TE**	**DO**	**NA**	**CIP**	**ENR**	**DA**	**SXT**	**AZM**

Bojanala	18	6 (33.3)	3 (16.7)	5 (27.8)	8 (44.4)	6 (33.3)	8 (44.4)	7 (38.9)	5 (27.8)	8 (44.4)	6 (33.3)	18 (100.0)	3 (16.7)	5 (27.8)	7 (38.9)	4 (22.2)	4 (22.2)
Dr. Kaunda	23	8 (34.8)	3 (13.0)	5 (21.7)	9 (39.1)	7 (30.4)	8 (34.8)	7 (30.4)	7 (30.4)	9 (39.1)	7 (30.4)	23 (100.0)	6 (26.1)	9 (39.1)	5 (21.7)	6 (26.1)	9 (39.1)
Subtotal	41	14 (34.1)	6 (14.6)	10 (24.4)	17 (41.5)	13 (31.7)	16 (39.0)	14 (34.1)	12 (29.3)	17 (41.5)	13 (31.7)	41 (100.0)	9 (22.0)	14 (34.1)	12 (29.3)	10 (24.4)	13 (31.7)
*P* value		0.9226	0.7446	0.6550	0.7318	0.8431	0.5291	0.5710	0.8528	0.7218	0.8431	NA	0.4696	0.4468	0.2310	0.7749	0.2482
Total	44	14 (31.8)	8 (18.2)	11 (25.0)	17 (38.6)	15 (34.1)	19 (43.2)	14 (31.8)	13 (29.5)	18 (40.9)	14 (31.8)	44 (100.0)	11 (25.0)	15 (34.1)	14 (31.8)	10 (22.7)	13 (29.5)

^a^Comprising three isolates of L. monocytogenes recovered.

^b^Consisting of 41 isolates of *Listeria* spp., other than *L. monocytogenes*, recovered.

### 3.7. Prevalence of Resistance Among *L. monocytogenes* and *Listeria* spp. by Type of Farm in Mpumalanga Province

Table 6 shows the prevalence of resistance among *L. monocytogenes* and *Listeria* spp. tested for isolates from Mpumalanga province, according to the farm type. Among the 12 isolates of *L. monocytogenes*, for the eight antimicrobial agents, the prevalence of resistance, ranging from 50% to 100%, was detected in six agents (CEF, TE, NA, CIP, ENR, and CLIN), 75% (6/8). No comparisons could be made among *L. monocytogenes* isolates recovered from a single farm type (feedlot).

**TABLE 6:** Antimicrobial resistance of isolates of *L. monocytogenes* and *Listeria* spp. recovered from farms to 16 antimicrobial agents by type of farm in Mpumalanga province.

Types of farms	No. of *L. monocytogenes* isolates tested	No. (%) of isolate^a^ resistant to:							
		CEF	CEP	TE	DOX	NA	CIP	ENR	CLIN
Feedlot	**12**	0 (0.0)	6 (50.0)	8 (66.7)	5 (41.7)	12 (100.0)	7 (58.3)	9 (75.0)	9 (75.0)

**Types of farms**	**No. of *Listeria* spp. isolates tested**	**CEF**	**CEP**	**TE**	**DOX**	**NA**	**CIP**	**ENR**	**CLIN**

Communal	13	1 (7.7)	10 (76.9)	1 (7.7)	3 (23.1)	13 (100.0)	6 (46.2)	7 (53.9)	6 (46.2)
Cow–calf operations	17	0 (0.0)	14 (82.4)	3 (17.7)	5 (29.4)	17 (100.0)	8 (47.1)	12 (70.6)	14 (82.4)
Feedlot	14	0 (0.0)	9 (64.3)	3 (21.4)	3 (21.4)	14 (100.0)	7 (50.0)	9 (64.3)	11 (78.6)
*P* value		0.4815	0.5033	0.6025	0.8618	NA	0.9778	0.6389	0.0711
Subtotal	44	1 (2.3)	33 (75.0)	7 (15.9)	11 (25.0)	44 (100.0)	21 (47.3)	28 (63.6)	31 (70.5)
Total	56	1 (1.8)	39 (69.6)	15 (26.8)	16 (24.6)	56 (100.0)	28 (50.0)	37 (66.1)	40 (71.4)

^a^All the isolates of *L. monocytogenes* and *Listeria* spp. were susceptible to penicillin, amoxicillin, ampicillin and sulfamethoxazole–trimethoprim, kanamycin, gentamycin, streptomycin, and AZI: azithromycin.

For the *Listeria* spp., resistance to antimicrobial agents within the farm types, for the prevalence of resistance ranging from 50% to 100%, was detected in three agents (CEP, NA, and ENR), 37.5% (3/8), in four agents (CEP, NA, ENR, and CLIN), 50% (4/8), and in five agents (CEP, NA, CIP, ENR, and CLIN), 62.5% (5/8) for communal, cow‐calf, and feedlot operations, respectively. A comparison of the prevalence of resistance to each of the eight antimicrobial agents for the *Listeria* spp. isolates recovered from the three types of farms showed no statistically significant differences (*p* > 0.05).

### 3.8. Prevalence of Resistance Among *L. monocytogenes* and *Listeria* spp. by the Type of Farm in North West Province

The prevalence of resistance to antimicrobial agents exhibited by *L. monocytogenes* and *Listeria* spp. according to the farm type in the North West Province as shown in Table 7. For the three isolates of *L. monocytogenes*, all recovered from a cow–calf operation, resistance was generally high across the 16 antimicrobial agents tested, with the prevalence of resistance ranging from 50% to 100% detected for six antimicrobial agents (AMC, CEF, S, NA, CIP, and CLIN), 37.5% (6/16). For *Listeria* spp., resistance levels were relatively low, with the majority in the 20%–40% range.

**TABLE 7:** Prevalence of antimicrobial resistance of *L. monocytogenes* and *Listeria* spp. was recovered in North West province according to the type of farm.

Type of farm	No. of *L. monocytogenes* isolates tested^a^	No. (%) of isolates resistant to:															
		P	AMC	AMP	CEP	CEF	S	CN	K	TE	DOX	NA	CIP	ENR	CLIN	SXT	AZM
Cow‐calf	3	0 (0.0)	2 (66.7)	1 (33.3)	0 (0.0)	2 (66.7)	3 (100.0)	0 (0.0)	1 (33.3)	1 (33.3)	1 (33.3)	3 (100.0)	2 (66.7)	1 (33.3)	2 (66.7)	0 (0.0)	0 (0.0)

**Type of farm**	**No. of *Listeria* spp. isolates tested^b^**	**No. (%) of isolates resistant to:**															
		**P**	**AMC**	**AMP**	**CEP**	**CEF**	**S**	**GEN**	**K**	**TE**	**DOX**	**NA**	**CIP**	**ENR**	**CLIN**	**SXT**	**AZM**

Communal	18	6 (33.3)	3 (16.7)	5 (27.8)	8 (50.0)	7 (38.9)	8 (50.0)	7 (38.9)	4 (22.2)	7 (38.9)	6 (33.3)	18 (100.0)	3 (16.7)	6 (33.3)	5 (27.8)	3 (16.7)	5 (27.8)
Cow‐calf	23	8 (34.8)	3 (13.0)	5 (21.7)	9 (39.1)	6 (26.1)	8 (34.8)	7 (30.4)	8 (34.8)	10 (43.5)	7 (30.4)	23 (100.0)	6 (26.1)	8 (34.8)	7 (30.4)	7 (30.4)	6 (26.1)
Subtotal	41	14 (34.1)	6 (14.6)	10 (24.4)	17 (41.5)	13 (31.7)	16 (39.0)	14 (34.1)	12 (29.3)	17 (41.5)	13 (31.7)	41 (100.0)	9 (22.0)	14 (34.1)	12 (29.3)	10 (24.4)	11 (26.8)
*p* value		0.9220	0.7446	0.6550	0.7318	0.3820	0.5291	0.5710	0.3804	0.7672	0.8431	NA	0.4700	0.9220	0.8528	0.3083	0.9035
Total	44	14 (31.8)	8 (18.2)	11 (25.0)	17 (38.6)	15 (34.1)	19 (43.2)	14 (31.8)	13 (29.5)	18 (40.9)	14 (31.8)	44 (100.0)	11 (25.0)	15 (34.1)	14 (31.8)	10 (22.7)	11 (25.0)

^a^Comprising 3 isolates of *L. monocytogenes* recovered.

^b^Consisting of 41 isolates of *Listeria* spp., other than *L. monocytogenes* recovered.

For the communal farms, resistance prevalence in the 20%–40% range was detected in 10 agents (P, AMP, CEF, GEN, K, TE, DOX, ENR, CLIN, and AZM), representing 62.5% (10/16) of the agents, compared with the 20%–40% range found in 13 agents (P, AMP, CEP, CEF, S, GEN, K, DOX, CIP, ENR, CLIN, SXT, and AZM), representing 81.3% (13/16) for the cow–calf operations. However, a comparison of the prevalence of resistance to each of the 16 antimicrobial agents between the communal and cow–calf operations showed no statistically significant differences (*p* > 0.05).

### 3.9. Prevalence of Resistance Among *L. monocytogenes* and *Listeria* spp. by and Type of Samples Collected From Farms in Mpumalanga Province

The distribution of resistance prevalence to eight antimicrobial agents among *L. monocytogenes* and *Listeria* spp. by the sample type in Mpumalanga province is shown in Table 8.

**TABLE 8:** Prevalence of resistance to antimicrobial agents among isolates of *L. monocytogenes* and *Listeria* spp. in Mpumalanga province by sample type.

Sample type**^a^**	**No. of *L. monocytogenes* isolates tested**	No. (%) of isolates**^b^** resistant to:							
		**CEF**	**CEP**	**TE**	**DOX**	**NA**	**CIP**	**ENR**	**CLIN**

Individual feces	7	0 (0.0)	3 (42.9)	5 (71.4)	3 (42.9)	7 (100.0)	5 (71.4)	6 (85.7)	4 (51.1)
Pooled feces	5	0 (0.0)	3 (60.0)	3 (60.0)	2 (40.00)	5 (100.0)	2 (40.0	3 (60.0)	5 (100.0)
*p* value		NA	0.5582	0.6788	0.9212	NA	0.2763	0.3105	0.2045
Subtotal	12	0 (0.0)	6 (50.0)	8 (66.7)	5 (41.7)	12 (100.0)	7 (58.3)	9 (75.0)	9 (75.0)

**Sample type**	**No. of *Listeria* spp. isolates tested**	No. (%) of isolates**^a^** resistant to:							
		**CEF**	**CEP**	**TE**	**DOX**	**NA**	**CIP**	**ENR**	**CLIN**

Effluent	1	0 (0.0)	1 (100.0)	0 (0.0)	0 (0.0)	1 (100.0)	0 (0.00	0 (0.0)	1 (100.0)
Individual feces	19	1 (5.3)	15 (79.0)	4 (21.1)	5 (26.3)	19 (100.0)	10 (52.6)	13 (68.4)	12 (63.2)
Pooled feces	12	0 (0.0)	7 (58.3)	3 (25.0)	3 (25.00)	12 (100.00	7 (58.3)	7 (58.3)	9 (75.0)
Feeds	6	0 (0.0)	4 (66.7)	0 (0.0)	2 (33.3)	6 (100.0)	2 (33.3)	5 (83.3)	3 (50.0)
Silage	5	0 (0.00	5 (100.0)	0 (0.0)	0 (0.0)	5 (100.0)	1 (20.0)	3 (60.0)	5 (100.0)
Water	1	0 (0.0)	1 (100.0)	0 (0.00	1 (100.0)	1 (100.0)	1 (100.0)	0 (0.0)	1 (100.0)
*p* value		1	0.2181	0.5147	0.4545	NA	0.1926	0.2881	0.2888
Subtotal	44	2 (2.3)	33 (75.0)	7 (15.9)	11 (25.0)	44 (100.00	21 (47.7)	28 (63.40	31 (70.5)
Total	56	2 (3.8)	39 (69.6)	15 (26.80	16 (28.6)	56 (100.0)	28 (50.00	37 (66.1)	40 (71.4)

^a^All the samples of effluents, feeds, silage, and water were negative for *L. monocytogenes*.

^b^All the isolates of *L. monocytogenes* and *Listeria* spp. were susceptible to penicillin, amoxicillin, ampicillin, and sulfamethoxazole–trimethoprim, kanamycin, gentamycin, streptomycin, and AZM: azithromycin.

For *L. monocytogenes* isolates, the prevalence of resistance to eight antimicrobial agents was relatively high, with resistance rates ranging from 50% to 100% per agent. For individual feces, a prevalence of resistance (50%–100%) was found to five antimicrobial agents (TE, NA, CIP, ENR, and CLIN), representing 62.5% (5/8). For the isolates from pooled feces, a similar pattern of five agents (CEP, TE, NA, ENR, and CLIN) was observed, with 62.5% (5/8) of isolates identified. However, the differences in the prevalence of resistance to the eight agents among *L. monocytogenes* isolates from individual and pooled feces were not statistically significant (*p* > 0.05).

Regarding the *Listeria* spp. isolates recovered from the six sample types, the number of agents to which 50%–100% resistance was detected was as follows: effluents, three agents (CEP, NA, and CLIN), 37.5% (3/8); individual feces, five agents (CEP, NA, CIP, ENR, and CLIN), 62.5% (5/8); pooled feces, five agents (CEP, NA, CIP, ENR, and CLIN), 62.5% (5/8); feeds, four agents (CEP, NA, ENR, and CLIN), 50% (4/8); silage, four agents (CEP, NA, ENR, and CLIN), 50% (4/8); and drinking water, five agents (CEP, DOX, NA, CIP, and CLIN), 62.5% (5/8). Similar patterns were observed across the sample types. Overall, the sample types did not significantly affect the prevalence of AMR among *Listeria* spp. (*p* > 0.05).

### 3.10. Prevalence of Resistance Among *L. monocytogenes* and *Listeria* spp. by the Type of Samples Collected From Farms in North West Province

The distribution of resistance prevalence among *L. monocytogenes* and *Listeria* spp. by the sample type in the North West province is shown in Table 9. Among the 3 *L. monocytogenes* isolates, resistance was the highest (50%–100%) to six agents (AMC, CEF, S, NA, CIP, and CLIN), with 37.5% (6/16) resistance. All isolates were susceptible to five antimicrobial agents (penicillin, cephalothin, gentamycin, sulfamethoxazole–trimethoprim, and azithromycin).

**TABLE 9:** Prevalence of antimicrobial resistance of *L. monocytogenes* and *Listeria* spp. isolates by type of sample in North West province.

		**No. (%) of isolates of *L. monocytogenes* resistant to:**															
Sample type**^a^**	**No. of isolates of *L.* *monocytogenes* tested**	**P**	**AMC**	**AMP**	**CEP**	**CEF**	**S**	**CN**	**K**	**TE**	**DOX**	**NA**	**CIP**	**ENR**	**CLIN**	**SXT**	**AZM**

Fecal	3	0 (0.0)	2 (66.7)	1 (33.3)	0 (0.0)	2 (66.7)	3 (100.0)	0 (0.0)	1 (33.3)	1 (33.3)	1 (33.3)	3 (100.0)	2 (66.7)	1 (33.3)	2 (66.7)	0 (0.0)	0 (0.0)

		**No. (%) of isolates of *Listeria* spp. resistant to:**															
Sample type**^b^**	**No. of other *Listeria* spp. isolates tested**	**P**	**AMC**	**AMP**	**CEP**	**CEF**	**S**	**GEN**	**K**	**TE**	**DOX**	**NA**	**CIP**	**ENR**	**CLIN**	**SXT**	**AZM**

Water	8	3 (37.5)	1 (12.5)	2 (25.0)	2 (25.0)	2 (25.0)	2 (25.0)	3 (37.5)	3 (37.5)	2 (25.0)	3 (37.5)	8 (100.0)	3 (37.5)	4 (50.0)	3 (37.5)	2 (25.0)	3 (37.5)
Feed	3	2 (66.7)	0 (0.0)	1 (33.3)	1 (33.3)	0 (0.0)	2 (66.7)	0 (0.0)	2 (66.7)	2 (66.7)	2 (66.7)	3 (100.0)	2 (66.7)	2 (66.7)	3 (100.0)	2 (66.7)	2 (66.7)
Fecal	30	9 (30.0)	5 (16.7)	7 (23.3)	14 (46.7)	11 (36.7)	12 (40.0)	11 (36.7)	7 (23.3)	13 (43.3)	8 (26.7)	30 (100.0)	4 (13.3)	8 (26.7)	8 (26.7)	6 (20.0)	8 (26.7)
*p* value		0.4316	0.1918	0.9278	0.5195	0.5343	0.4412	0.4909	0.2467	0.4228	0.3380	NA	0.0515	0.2175	0.5403	0.1996	0.3380
Subtotal	41	14 (34.1)	6 (14.6)	10 (21.4)	17 (41.5)	13 (31.7)	16 (39.0)	14 (34.1)	12 (29.3)	17 (41.5)	13 (31.7)	41 (100.0)	9 (22.0)	14 (34.1)	12 (29.3)	10 (24.4)	13 (31.7)
Total	44	14 (31.8)	8 (18.2)	11 (25.0)	17 (38.6)	15 (34.1)	19 (43.2)	14 (31.8)	13 (29.5)	18 (40.9)	14 (31.8)	44 (100.0)	11 (25.0)	15 (34.1)	14 (31.8)	10 (22.7)	13 (29.5)

^a^No isolate of *L. monocytogenes* was recovered from feed, water, silage, and effluent samples.

^b^No isolate of *Listeria* spp. was isolated from silage and effluent samples.

The prevalence of resistance to antimicrobial agents was generally low (20%–40%) for *Listeria* spp. isolates from water as found in 13 agents (P, AMP, CEP, CEF, S, CN, K, TE, DOX, CIP, CLIN, SXT, and AZM), 81.3% (13/16) and fecal isolates with a range of resistance (20%–40%) detected in 11 agents (P, AMP, CEF, S, CN, K, DOX, ENR, CLIN, SXT, and AZM), 68.8% (11/16). However, for isolates from feed, the prevalence of resistance was high (50%–100%), as detected in 11 agents (P, S, K, TE, DOX, NA, CIP, ENR, CLIN, SXT, and AZM), corresponding to 68.8% (11/16). Overall, differences in the prevalence of resistance to the 16 antimicrobial agents across the three sample types (water, feed, and feces) were not statistically significant (*p* > 0.05).

### 3.11. Frequency of Resistance Patterns Exhibited by Isolates of *L. monocytogenes* and *Listeria* spp. Recovered From Cattle Farms in Mpumalanga Province

Among the 12 *L. monocytogenes* isolates from Mpumalanga province, 10 AMR patterns were observed, of which 9 (90%) were multidrug‐resistant (MDR), i.e., exhibiting resistance to at least three antimicrobial classes. The range of isolates involved in MDR was 3 (2 isolates) to 7 (2 isolates) (Table 10).

**TABLE 10:** Resistance patterns exhibited by *Listeria monocytogenes and Listeria* spp. isolated from cattle farm sources in Mpumalanga province.

**Resistance patterns of *L. monocytogenes***	No. (%)**^a^** of isolates exhibiting resistance patterns	**No. of agents involved in the pattern**

TE‐DOX‐NA‐CIP‐ENR‐CLIN	2 (16.7)	6
CEP‐TE‐DOX‐NA‐CIP‐ENR‐CLIN	2 (16.7)	7
CEP‐NA‐CLIN	1 (8.3)	3
TE‐NA‐CLIN	1 (8.3)	3
CEP‐NA‐ENR‐CLIN	1 (8.3)	4
CEP‐NA‐CIP‐ENR	1 (8.3)	4
CE‐NA	1 (8.3)	2
TE‐NA‐CIP‐ENR	1 (8.3)	4
TE‐DOX‐NA‐CIP‐ENR‐CLIN	1 (8.3)	6
TE‐NA‐ENR‐CLIN	1 (8.3)	4

**Resistance patterns of *Listeria* spp.**	No. (%)**^b^** of isolates exhibiting resistance patterns	**No. of agents involved in the pattern**

CEP‐NA‐ENR‐CLIN	8 (18.2)	4
CEP‐NA‐CLIN	5 (11.4)	3
CEP‐NA‐CIP‐ENR	4 (9.1)	4
CEP‐NA‐CIP‐ENR‐CLIN	3 (6.8)	5
CEP‐TE‐DOX‐NA‐ENR‐CLIN	3 (6.8)	6
CEP‐NA‐CIP‐CLIN	2 (4.6)	4
CEP‐TE‐NA‐CIP‐CLIN	2 (4.6)	5
TE‐DOX‐NA‐CIP‐ENR‐CLIN	2 (4.6)	6
CEP‐DOX‐NA‐ENR‐CLIN	1 (2.3)	5
CEP‐DOX‐NA‐CIP‐CLIN	1 (2.3)	5
CEP‐NA‐CIP	1 (2.3)	3
NA‐CIP‐ENR	1 (2.3)	3
CEP‐CEF‐NA‐CIP‐ENR	1 (2.3)	5
NA‐ENR	1 (2.3)	2
DOX‐NA	1 (2.3)	2
CEP‐DOX‐NA	1 (2.3)	3
DOX‐NA‐ENR	1 (2.3)	3
CEP‐NA	1 (2.3)	2
NA‐CIP	1 (2.3)	2
NA‐CLIN	1 (2.3)	2
NA‐CIP‐CLIN	1 (2.3)	3
NA‐CIP‐ENR‐CLIN	1 (2.3)	4
DOX‐NA‐CIP‐ENR‐CLIN	1 (2.3)	5

^a^Based on 12 isolates of *L. monocytogenes*.

^b^Based on 44 isolates of *Listeria* spp.

For the 44 isolates of other *Listeria* spp., 23 resistance patterns were observed, of which 18 (78.3%) were MDR. The range of isolates exhibiting MDR was 3 (two isolates) to 6 (five isolates).

The frequency of MDR in *L. monocytogenes* (90%; 9 of 10) and in *Listeria* spp. (78.3%; 18/23) was not statistically significantly different (*p* = 0.4217).

## 4. Discussion

The data obtained from our cross‐sectional studies, conducted concurrently in two provinces (Mpumalanga and North West) of the nine provinces in South Africa, on the prevalence of resistance to 16 antimicrobial agents, including some used in veterinary and human practices in the country, in *L. monocytogenes* and *Listeria* spp. recovered from cattle farms provide invaluable information on the AMR profiles of *Listeria* recovered from these sources [1, 2] [52]. Our study is also significant for providing information on the distribution of AMR *L. monocytogenes* strains, as well as potential factors (geographic farm location, farm type, and sample type) within and between the two provinces. The possibility of AMR *L. monocytogenes* from cattle farms can spill over into the human food chain through consumption of contaminated beef from slaughtered cattle has been reported by others in South Africa, Jordan, and Iraq [21, 49, 56–58, 64, 65].

It is of therapeutic concern that across cattle farms sampled in the Mpumalanga and North West provinces, all 100 isolates of *L. monocytogenes* and *Listeria* spp. exhibited resistance to at least one of the 16 antimicrobial agents tested in agreement with published reports [21, 66–68]. Importantly, some of these antimicrobials are routinely used as frontline or second‐line treatments for human listeriosis [4, 10–12]. It is concerning to observe that, regardless of the sources/types of samples collected from farms, among resistant strains of *L. monocytogenes*, which were resistant to 11 (68.8%) of the 16 agents, the prevalence of resistance ranged from 41.7% (doxycycline) to 100% (cefotaxime, kanamycin, streptomycin, azithromycin, and nalidixic acid), and for *Listeria* spp., which were resistant to 8 (50%) of 16 agents, the prevalence of resistance ranged from 2.3% (cefotaxime) to 100% (nalidixic acid) in Mpumalanga province. However, in the North West province, isolates of *L. monocytogenes* were resistant to 11 (68.8%) of 16, ranging from 33.3% (kanamycin) to 100% (streptomycin, ampicillin, and nalidixic acid), and among *Listeria* spp. resistant to 100% (16/16) agents, ranging from 9.5% (kanamycin) to 100% (nalidixic acid). These findings indicate widespread resistance of 68.8%–100% to the 16 agents tested in *L. monocytogenes* and *Listeria* spp., with implications for the treatment of infections caused by *Listeria* and other pathogens on cattle farms in both provinces, suggesting differences in antimicrobial use between the provinces. Studies by others have reported a variation in the frequency of AMR among *L. monocytogenes* and *Listeria* spp. isolates from cattle and farm environments in the country and elsewhere, attributable to the misuse of antimicrobials on livestock farms [21, 26, 64–67, 69]. It is noteworthy that all isolates of *L. monocytogenes* in Mpumalanga Province exhibited resistance to five antimicrobials (cefotaxime, kanamycin, streptomycin, nalidixic acid, and azithromycin), compared to only three (streptomycin, nalidixic acid, and ampicillin) in North West province, reflecting similarities and differences between the two provinces. The differences in AMR prevalence between the two provinces may be attributable to the misuse of antimicrobials on livestock farms, as reported elsewhere [21, 26, 64, 65, 67, 69]. It is also pertinent to note that all *L. monocytogenes* isolates recovered from the two provinces were resistant to streptomycin and nalidixic acid. Of therapeutic relevance is that *L. monocytogenes* isolates in both provinces exhibited resistance to six antimicrobial agents, including ampicillin, found only in the North West province. This is because five of these antimicrobial agents are not considered frontline therapeutic options for human listeriosis, as reported for penicillin, ampicillin, and others [4, 10, 11]. However, of significance is our finding that although all the isolates of *L. monocytogenes* in North West province were resistant to ampicillin, which is therapeutically important in human listeriosis [4, 10, 69, 70], all these ampicillin‐resistant isolates of *L. monocytogenes* were also susceptible to penicillin, gentamycin, and sulfamethoxazole–trimethoprim, thus posing no therapeutic threat.

A therapeutic advantage is that all isolates of *L. monocytogenes* recovered from cattle farms in Mpumalanga province were susceptible to five antimicrobial agents (penicillin, ampicillin, amoxicillin–clavulanate acid, gentamycin, and sulfamethoxazole–trimethoprim), and in North West province, all were susceptible to penicillin, gentamycin, sulfamethoxazole–trimethoprim, cephalothin, and azithromycin. This is because some of these antimicrobial agents have been proven effective in the treatment of human listeriosis, some as frontline agents [4, 10–12]. Therefore, the data suggest prudent use of these therapeutically important antimicrobial agents on cattle farms in both provinces. However, the prevalence of resistance to specific antimicrobial agents varies among *L. monocytogenes* isolates recovered from cattle farms elsewhere; for example, in a study of cattle farms in Egypt [26], all *L. monocytogenes* strains from dairy farms were resistant to penicillin, neomycin, cefoxitin, and nalidixic acid. They were also resistant to amoxicillin, cloxacillin, cefotaxime, amikacin, erythromycin, norfloxacin, tetracyclines, and gentamicin, which are frequently valuable for the treatment of human listeriosis. On the contrary, Obaidat [21] reported that more than 90% of *L. monocytogenes* isolates from imported beef cattle in Jordan were resistant to ampicillin, penicillin, and erythromycin; in Greece, in the cattle isolates of *L. monocytogenes*, Tsitsos et al. [71] reported the highest resistance rate for clindamycin, followed by vancomycin, tetracycline, and ciprofloxacin; however, all strains were susceptible to amoxicillin/clavulanate, ampicillin, chloramphenicol, erythromycin, gentamicin, meropenem, penicillin, rifampicin, and trimethoprim–sulfamethoxazole; and in Japan, among the *L. monocytogenes* isolates from cattle, Hasegawa et al. [27] reported that isolates of *L. monocytogenes* in Japan were susceptible to penicillin, ampicillin, amoxicillin, gentamicin, kanamycin, streptomycin, erythromycin, vancomycin, tetracycline, chloramphenicol, ciprofloxacin, and trimethoprim/sulfamethoxazole, which agree with the findings regarding four agents found in our study. It cannot be overemphasized that differences in resistance profiles to individual antimicrobial agents across countries reflect a variation in their use.

Clinically, it is relevant that all our *L. monocytogenes* isolates recovered from Mpumalanga province were susceptible to both penicillin and ampicillin. In contrast, in North West province, although all isolates were susceptible to penicillin, they were all resistant to ampicillin. This is of therapeutic importance, since both antimicrobials are typically used as frontline treatment options [4, 10–12]. The findings may partly be explained by the difference in the use of ampicillin (dose and frequency) on cattle farms in both provinces. However, the potential adverse therapeutic effects of the findings may be reduced, as all our AMP‐resistant *L. monocytogenes* isolates from the North West province were also penicillin‐susceptible. It has been documented that *L*. *monocytogenes* can exhibit different susceptibilities to penicillin and ampicillin and that, although susceptibility is generally high for both, they are distinct compounds. Studies have shown variations in resistance rates, inhibitory concentrations, and clinical effectiveness [21, 64, 72].

It is important to note that resistance to the Tetracycline class (tetracycline and doxycycline) was observed in *L. monocytogenes* and *Listeria* spp. in both provinces, with a relatively high frequency of 61%. This high tetracycline resistance frequency ranked third, behind fluoroquinolones (100%) and cephalosporins (74%) and is widely used across livestock farms in South Africa. The observed high prevalence of resistance to tetracyclines may be partly attributed to the South African government’s policy, which legalized the OTC availability of specific antimicrobial agents, such as tetracyclines, sulfonamides, cloxacillin intramammary, fosfomycin, tylosin, and kitasamycin, to facilitate the timely treatment of easily recognizable endemic diseases. The policy has enabled livestock farmers to obtain tetracyclines at comparatively low cost, which may have contributed to their widespread use, thereby increasing the relatively high prevalence of tetracycline resistance among *L. monocytogenes* and *Listeria* spp. in the current study. Several studies in South Africa have similarly reported a high prevalence of tetracycline resistance in *L. monocytogenes* and *Listeria* spp. in RTE beef products [49], *Salmonella* spp. from beef abattoirs and retail outlets, and chickens from the informal market [73, 74], Shiga toxin–producing *Escherichia coli* from beef abattoirs and retail outlets [75], and *Campylobacter* spp. isolates from chickens [76]. These indicate a widespread national problem of tetracycline resistance in the country’s livestock industry. It is therefore imperative for the government to review the OTC availability of tetracyclines to livestock farmers to decrease the prevalence of AMR in *Listeria* and other bacterial pathogens in both provinces and across South Africa.

The distribution of resistance to antimicrobial agents among *L. monocytogenes* and *Listeria* spp. from cattle farms in Mpumalanga and North West provinces to antimicrobial classes was found to be statistically significantly lower for beta‐lactams (17%) and sulfonamides (6%) than for the other six classes of antimicrobial agents (24%–100%), a finding expected based on the level of resistance observed to individual antimicrobials. This is because penicillin, amoxicillin–clavulanic acid, and sulfamethoxazole–trimethoprim are antimicrobials in these two classes and have been reported to be efficacious in treating listeriosis by others. The findings also indicate a judicious use of these antimicrobial agents on cattle farms in both provinces.

MDR *L. monocytogenes* strains in livestock and meat retailing have been documented to pose serious therapeutic challenges for consumers of meat and meat products in the country [39, 40, 43, 44, 56, 57]. It is therefore not surprising that in Mpumalanga province, 10 AMR patterns were detected in *L. monocytogenes* isolates, of which 90% were MDR. The potential therapeutic implications are particularly evident given the range of agents involved (3–7), which can broaden the spectrum of AMR. In South Africa, a similarly high prevalence of MDR *L. monocytogenes* in beef and beef products, ranging from 50% to 100%, has been reported [46, 49, 57]. Similar or higher frequencies of resistance patterns (4–25) and MDR (96.9% and 98.1%) in *L. monocytogenes* have been reported in Jordan and Egypt [21, 26, 68]. Equally important, among the nine MDR isolates in *L. monocytogenes*, tetracyclines, doxycycline, and ciprofloxacin were detected in six, three, and five isolates, respectively. It is worth noting that Tetracycline and Doxycycline have been reported to be effective against *L. monocytogenes* infections. However, they are often second‐line treatments due to emerging resistance. [4]. It has also been documented that Ciprofloxacin is used to treat listeriosis, along with other antibiotics such as ampicillin, but it is often used in combination or for specific situations, as its efficacy can vary, with reported ciprofloxacin resistance in *L. monocytogenes* [76, 77]. It is important to emphasize the therapeutic importance of the risk of complications posed by MDR *L. monocytogenes* from cattle farms entering the human food chain via abattoirs and retail outlets. Our detection of 23 resistance patterns, of which 18 (78.3%) were MDR, in the 44 *Listeria* spp. isolates, consisting of resistance to tetracycline (three isolates), doxycycline (seven) isolates, and ciprofloxacin (13 isolates) is relevant and cannot be ignored, considering that, although they are nonpathogenic, they can transfer resistance genes or plasmids to important pathogens for bovine and human listeriosis, such as *L. monocytogenes* and *L. ivanovii* [4, 5, 9]. Our findings on the frequency of MDR in *Listeria* isolated from cattle farms in the two provinces may reflect pressure from overuse, driven by the OTC availability and overprescription of antibiotics in veterinary and human medicine in the country. Their widespread use as growth promoters and prophylactics and in livestock therapy may have contributed to the increased prevalence of bacterial resistance [41].

The overall high prevalence and antimicrobial agent‐specific frequency of resistance to antimicrobial agents and MDR in 100 *L. monocytogenes* and *Listeria* spp. isolates from cattle farms in Mpumalanga and North West provinces provide evidence of inadequate control of antimicrobial use in the country’s cattle production system. The overwhelming high prevalence of resistance to several antimicrobial agents in *L. monocytogenes* and *Listeria* spp. recovered from cattle, beef, and beef products, as similarly documented in other pathogens, such as *Salmonella spp*., Shiga‐toxin producing *Escherichia coli* (STEC), and *Campylobacter spp*., in the country [44–46, 49, 50, 64, 73–76] the gravity of the AMR and MDR problem in the country, more so that transfer of intergenus and intraspecies of AMR plasmids and genes have been reported in bacteria in the literature [77–79].

It is worth noting that the South African government has been aware of the threat of AMR, which has led to the development of a five‐strategic framework to control the prevalence of AMR nationally [36, 37]. However, implemented legislation and policies to control AMR in bacterial pathogens in the country have been unsuccessful to date. The outcome of this initiative is that the least expensive OTC antimicrobial agents are widely used without veterinary oversight [42, 43]. The high prevalence of resistance to antimicrobial agents and the frequency of MDR across some antimicrobials in *L. monocytogenes* and *Listeria* spp. in the two provinces may therefore be an outcome of ineffective AMR control policy, with therapeutic implications. The endemic high prevalence of AMR in *Listeria* and other pathogens should therefore serve as an incentive for the government of South Africa to embrace and implement the One Health approach, which has been proven to be an effective strategy for controlling AMR in pathogens [33, 80, 81], thus providing benefits to food safety and therapy. This is because, globally, particularly in developed countries, the trend is to implement the One Health approach to control AMR in *Listeria* by introducing several preventive measures [30–33]. This approach must focus on reducing agricultural antibiotic use, improving food safety protocols to prevent environmental contamination, and monitoring transmission pathways to manage risks [34, 35]. It cannot be overemphasized that the One Health approach should be implemented holistically to be successful. The need for South Africa’s government to implement the One Health approach promptly cannot be ignored.

The potential effects of the geographic locations of cattle farms from where the isolates of *L. monocytogenes* and *Listeria* spp. on the prevalence of AMR are evident within Mpumalanga province, where cattle farm location significantly affected the prevalence of ciprofloxacin‐resistant *L. monocytogenes*. However, our study found that in the North West province, the geographic location of the cattle farm did not significantly affect the prevalence of resistance to the 16 antimicrobial agents tested. The significantly higher prevalence of ciprofloxacin resistance in *L. monocytogenes* isolates in the Emalahleni district than in the Delma district may be partly due to the differences in the numbers of *L. monocytogenes* tested in Mpumalanga province (*n* = 12) and North West province (*n* = 3), as well as differences in antimicrobial use between the districts. Reports by others indicate that geographic location significantly affects the distribution of AMR in *L. monocytogenes* and other bacterial pathogens [27, 29]. A future questionnaire survey of cattle farmers and veterinarians on the use of different antimicrobial agents in the Emalahleni district of Mpumalanga province may provide additional information to explain our findings.

Considering another level of geographic effect on the prevalence of AMR, it is interesting that, regardless of the sources (district, farm type, and sample type) of the *L. monocytogenes* isolates, a comparison of overall AMR prevalence revealed statistically significant differences between Mpumalanga and North West provinces, demonstrating another potential geographic effect, albeit interprovincial. This is reflected in significant differences in the prevalence of resistance to Amoxycillin‐Clavulanic Acid, Kanamycin, and Azithromycin, suggesting differences in the use of the three antimicrobial agents between the two provinces and a geographic location effect. Equally significant but contrary to the prevalence of resistance to some antimicrobial agents, the same prevalence of resistance (0%), i.e., 100% susceptibility, was observed in *L. monocytogenes* for five antimicrobial agents (penicillin, gentamycin, sulfamethoxazole–trimethoprim, streptomycin, and nalidixic acid), three of which are among the frontline agents used for treating human listeriosis [4, 10–12]. The inference is that, despite potential differences in antimicrobial use practices between the two provinces investigated, prudent use of penicillin, gentamycin, and sulfamethoxazole–trimethoprim, which are known effective agents for treating human listeriosis [4, 10–12], appears to be observed.

Notably, the other variables (farm type and sample type) investigated did not significantly affect the prevalence of AMR among *L. monocytogenes* and *Listeria* spp., a finding inconsistent with some published reports [26–29]. Unlike in our study, which failed to detect any statistically significant difference in the prevalence of resistance among *L. monocytogenes* and *Listeria* spp. across the three cattle farms (communal, cow‐calf, and feedlot operations) in both provinces, it has been documented that cattle farm type significantly influences AMR in *Listeria* spp. This has been attributed to differences in management systems, including intensification, animal sources, and the use of antimicrobial agents (prophylaxis, growth promoters, and therapy) [15, 16], as well as to hygienic practices and other factors [82–84]. Our findings may, in part, be due to the relatively high prevalence of resistance across the three farm types and to the disproportionate number of isolates tested.

Some of the unavoidable limitations of this study include the following: the small number of isolates of *L. monocytogenes*, 12 and 3, from Mpumalanga and North West province, respectively; the disproportionate types and small number of isolates available for AMR determination among the three variables (district, farm type, and sample type) in Mpumalanga and North West provinces, to investigate their potential significant impact on the prevalence of resistance among *L. monocytogenes* and *Listeria* spp.; and the unfortunate irretrievable data on the MDR in the isolates of *L. monocytogenes* and *Listeria* spp.

## 5. Conclusion

Our findings showed that 100 *Listeria* isolates exhibited resistance to at least one of the 16 antimicrobial agents, regardless of antimicrobial type, and had AMR frequencies ranging from 68.8% to 100% among *L. monocytogenes* and *Listeria* spp. isolates recovered from both Mpumalanga and North West provinces provide evidence of potential therapeutic implications for their use in cattle and humans. The potential of therapeutic threat is further raised by the fact that among *L. monocytogenes* isolates from Mpumalanga province, the prevalence of resistance ranged from 41.7% (Doxycycline) to 100% (cefotaxime, kanamycin, streptomycin, and nalidixic acid), compared to from 33.3% (kanamycin, tetracycline, doxycycline, nalidixic acid, and enrofloxacin) to 100% (streptomycin, nalidixic acid, and ampicillin) in the North West province. The findings that all *L. monocytogenes* isolates recovered from both provinces were susceptible to five antimicrobial agents (penicillin, ampicillin, amoxicillin–clavulanate, gentamicin, and sulfamethoxazole–trimethoprim), which are used as first‐ or second‐line agents for human listeriosis, suggest prudent use of these important agents in both provinces. The ability of antimicrobial‐resistant *Listeria* spp. and *L. monocytogenes* to transfer resistance genes or plasmids intraspecies increases the risk of transfer from nonpathogenic to pathogenic *L. monocytogenes*. The overall high resistance to antimicrobial agents in *L. monocytogenes* and *Listeria* spp. in both provinces in South Africa is therefore indicative of a need for the South African government to review the policy that permits the OTC availability of specific antimicrobial agents, such as tetracyclines, and the uncontrolled use of antibiotics for prophylaxis, as growth promoters, and in the treatment of animal and human diseases. Finally, it is important for the South African government to implement the One Health approach holistically, as a matter of urgency, to address the high AMR problem. Removing policies that are counterproductive to reducing AMR in the country’s livestock industry cannot be overemphasized.

## Author Contributions

A.A.A. and N.G. conceived and designed the research. A.A.A., N.G., and R.M. secured funding, administered the project, and supervised it. K.C.M. and N.C.M. collected samples and conducted laboratory analyses, and Y.B.N., K.C.M., N.C.M., and A.A.A. analyzed the data. K.C.M., N.C.M., and A.A.A. wrote the first draft of the manuscript.

## Funding

Open access funding was provided by the University of Pretoria. This study was funded by the Red Meat Research and Development, South Africa (RMRD‐SA) under Grant No.: 2018–12‐20, December 2018, which enabled us to conduct the study.

## Disclosure

All authors read and approved the final version the manuscript.

## Ethics Statement

Before the commencement of the study, approvals were obtained from the following bodies and committees: Research Ethics Committee (REC) of the Faculty of Veterinary Science, University of Pretoria, South Africa (*REC 138–19*), Animal Ethics Committee (AEC) of the Faculty of Veterinary Science, University of Pretoria, South Africa (*REC 138–19*), issued on November 04, 2019, and Section 20 from the Department of Agriculture, Forestry and Fisheries (DAFF) [No.:12/11/1/1/8(1131)], South Africa.

## Consent

Informed consent was obtained from managers or owners of cattle farms in Mpumalanga and North West provinces, from which samples were collected. This was achieved by visiting the farmers to provide information on the project, including the types of samples to be collected, the confidentiality of their participation, and the results of the samples.

## Conflicts of Interest

The authors declare no conflicts of interest.

## Data Availability

The data supporting this study’s findings are available at upspace@up.ac.za and http://repository.up.ac.za, reference number 4870.
